# Alternate-Day Versus Consecutive-Day Anti-T Lymphocyte Globulin Induction in First Living-Donor Kidney Transplant Recipients at Low Immunological Risk: A Retrospective Cohort Study

**DOI:** 10.3390/jcm15197348

**Published:** 2026-09-22

**Authors:** Gulay Yilmaz, Ebru Ozdemir, Abdulhak Hamit Karayagiz, Ibrahim Berber, Ulkem Cakir

**Affiliations:** 1 Department of Nephrology, Basaksehir Cam and Sakura City Research and Training Hospital, Istanbul 34480, Turkey; 2 Organ Transplantation Center, Acibadem Atasehir Hospital, Istanbul 34642, Turkey; 3 Department of General Surgery, Esenyurt Necmi Kadioglu State Hospital, Istanbul 34517, Turkey; 4 Organ Transplantation Center, Acibadem University, Istanbul 34638, Turkey; 5 Department of Nephrology, Acibadem University, Istanbul 34638, Turkey

**Keywords:** kidney transplantation, anti-T lymphocyte globulin, induction therapy, living donor, bacterial infection, immunosuppression, clinical outcomes

## Abstract

**Background/Objectives:** Anti-T lymphocyte globulin (ATLG) is widely used as induction therapy in kidney transplantation; however, the clinical impact of the interval between individual ATLG doses remains poorly defined. We compared alternate-day and consecutive-day ATLG administration in first living-donor kidney transplant recipients at low immunological risk. **Methods:** This retrospective single-center cohort study included 195 first living-donor kidney transplant recipients who received ATLG induction therapy (alternate-day, *n* = 97; consecutive-day, *n* = 98). All recipients underwent their first kidney transplantation, received grafts from related living donors, were negative for donor-specific antibodies before transplantation, and received standardized maintenance immunosuppression. ATLG was initiated at approximately 2.0–2.5 mg/kg per administration, with subsequent doses individualized according to peripheral CD3+ T-cell and lymphocyte counts. The primary outcome was the occurrence of at least one bacterial infection during the first post-transplant year. Secondary outcomes included biopsy-proven acute rejection, BK virus and cytomegalovirus (CMV) positivity, graft function, graft loss, and patient survival; rejection, graft loss, and mortality were recorded throughout the available follow-up. Multivariable logistic regression analysis was performed to identify independent predictors of bacterial infection. **Results:** Baseline recipient and donor characteristics were generally comparable between groups, although recipients receiving consecutive-day ATLG had higher rates of PRA class I and II positivity. Cumulative weight-adjusted ATLG exposure was comparable between the alternate-day and consecutive-day groups (7.4 [7.1–7.8] vs. 7.2 [5.4–10.2] mg/kg, *p* = 0.207). The incidence of bacterial infection during the first post-transplant year was virtually identical in the alternate-day and consecutive-day groups (50.5% vs. 50.0%, *p* = 1.000). Likewise, no significant between-group differences were observed in biopsy-proven acute rejection (2.1% vs. 8.2%, *p* = 0.100), BK virus positivity (18.6% vs. 14.3%, *p* = 0.446), CMV positivity (17.5% vs. 12.2%, *p* = 0.321), graft loss (4.1% vs. 4.1%, *p* = 1.000), or all-cause mortality (7.2% vs. 4.1%, *p* = 0.372). In multivariable analysis, the ATLG administration schedule was not independently associated with bacterial infection (adjusted OR 1.21, 95% CI 0.64–2.30, *p* = 0.560). Recipient body mass index was the only independent predictor of bacterial infection (adjusted OR 1.08 per kg/m^2^, 95% CI 1.00–1.15, *p* = 0.041). **Conclusions:** Among first living-donor kidney transplant recipients at low immunological risk, alternate-day ATLG administration was associated with a similar incidence of bacterial infection during the first post-transplant year. When cumulative weight-adjusted ATLG exposure was accounted for, the interval between individual doses was not independently associated with bacterial infection. Crude secondary-outcome comparisons should be interpreted cautiously because overall follow-up differed between groups. These findings support further prospective evaluation of flexible ATLG administration schedules in carefully selected kidney transplant recipients.

## 1. Introduction

Kidney transplantation is the preferred kidney replacement therapy for suitable patients with end-stage kidney disease because it is associated with lower mortality and better health-related quality of life than maintenance dialysis [1,2]. Despite substantial advances in surgical techniques and maintenance immunosuppression, acute rejection and infectious complications remain major causes of post-transplant morbidity. Consequently, induction immunosuppressive therapy is widely used to reduce the risk of early rejection, with the choice of induction agent guided by the recipient’s immunological risk and the expected balance between efficacy and treatment-related toxicity [3].

Anti-T lymphocyte globulin (ATLG; Grafalon^®^, Neovii Biotech, Graefelfing, Germany) is a rabbit-derived polyclonal lymphocyte-depleting antibody preparation widely used for induction therapy in kidney transplantation. Unlike conventional rabbit anti-thymocyte globulin produced by immunization with human thymocytes, ATLG is generated using the Jurkat T-lymphoblast cell line, resulting in a distinct antibody profile and immunological characteristics [4]. Its principal mechanism of action is profound T-cell depletion accompanied by modulation of several immune pathways involved in allograft rejection [4,5]. Although highly effective, ATLG may also increase the risk of bacterial, viral, and opportunistic infections as well as hematological adverse effects [6,7].

Current clinical practice increasingly individualizes ATLG dosing according to recipient body weight, with cumulative exposure commonly reported as mg/kg rather than as an absolute dose [6,7]. Recent studies have shown that cumulative weight-adjusted ATLG exposure influences both efficacy and safety, whereas excessive exposure may increase the risk of infectious complications [8]. However, despite growing attention to cumulative ATLG exposure, the clinical importance of the interval between individual ATLG doses remains poorly understood.

Alternative ATLG administration schedules are frequently adopted in routine practice because of hematological toxicity, infusion-related adverse events, delayed postoperative recovery, or logistical considerations. Nevertheless, previous studies have generally included heterogeneous populations with different donor types, immunological risk profiles, donor-specific antibody (DSA) status, previous transplant histories, and maintenance immunosuppressive regimens [3,6,7,9]. Consequently, it remains uncertain whether clinical outcomes are influenced by the timing of ATLG administration itself or by differences in baseline risk and cumulative immunosuppressive exposure.

To minimize these potential sources of confounding, we evaluated a homogeneous cohort consisting exclusively of first living-donor kidney transplant recipients with low immunological risk. All recipients were undergoing their first kidney transplantation, were negative for donor-specific antibodies before transplantation, and received standardized maintenance immunosuppression with tacrolimus, mycophenolate mofetil, and corticosteroids. Cumulative ATLG exposure was expressed as total administered dose per kilogram of recipient body weight, enabling evaluation of the administration schedule while accounting for overall drug exposure.

However, a specific knowledge gap remains: it is unclear whether the interval between individual ATLG doses independently influences clinical outcomes when cumulative weight-adjusted exposure and maintenance immunosuppression are comparable. ATLG induces sustained peripheral T-cell depletion that persists beyond administration of an individual dose. Therefore, when subsequent dosing is guided by peripheral T-cell/lymphocyte depletion and cumulative weight-adjusted exposure is maintained, modest extension of the interval between individual doses may not necessarily reduce the overall pharmacodynamic effect. This provided the pharmacological rationale for evaluating an alternate-day administration strategy.

We aimed to compare alternate-day and consecutive-day ATLG induction in first living-donor kidney transplant recipients with low immunological risk and to determine whether dose spacing was associated with clinically relevant outcomes when cumulative weight-adjusted ATLG exposure was accounted for. The primary outcome was the occurrence of at least one clinically documented bacterial infection requiring antimicrobial treatment during the first post-transplant year. Secondary outcomes included biopsy-proven acute rejection, delayed graft function, BK virus positivity, cytomegalovirus positivity, graft function, graft loss, and patient survival.

The study was not designed to demonstrate superiority, equivalence, or non-inferiority of either schedule, but rather as a real-world comparative assessment of two institutional dosing schedules. Accordingly, the findings were interpreted as hypothesis-generating rather than as evidence of definitive equivalence or cost-effectiveness.

## 2. Materials and Methods

### 2.1. Study Design

This retrospective single-center cohort study was conducted at the Organ Transplantation Center of Acibadem International Hospital, Istanbul, Türkiye. The study protocol was approved by the Ethics Committee of Acibadem University (Approval No. 2021-15/23; 12 August 2021) and was conducted in accordance with the ethical principles of the Declaration of Helsinki. Written informed consent for kidney transplantation and the use of anonymized clinical data for scientific research had been obtained from all recipients before transplantation. Recipients were assigned to treatment groups according to the institutional induction protocol in effect at the time of transplantation.

During the initial study period, consecutive-day anti-T lymphocyte globulin (ATLG) administration was the standard induction regimen. Following an institutional protocol modification, alternate-day ATLG administration became the standard induction strategy for all subsequently transplanted recipients. Treatment allocation was therefore determined exclusively by the timing of transplantation and institutional practice rather than by recipient characteristics or physician preference. The transition to alternate-day ATLG administration was a general institutional protocol change intended to provide greater dosing flexibility, improve the practical tolerability of the induction regimen, and facilitate treatment logistics. It was not implemented selectively because of postoperative renal dysfunction and was not intended as a strategy to delay calcineurin inhibitor initiation.

### 2.2. Study Population

Adult recipients undergoing first living-donor kidney transplantation between 2017 and 2021 were eligible for inclusion. To obtain a homogeneous study population, only recipients with low immunological risk were included. All recipients underwent their first kidney transplantation, received grafts exclusively from related living donors, and were negative for donor-specific antibodies (DSA) before transplantation. Recipients undergoing deceased-donor transplantation, retransplantation, or those with incomplete clinical records were excluded. As this was a retrospective cohort study, all consecutive recipients who fulfilled the predefined eligibility criteria during the study period were included; therefore, no a priori sample-size calculation was performed.

A total of 195 recipients fulfilled the eligibility criteria. Ninety-seven recipients received alternate-day ATLG induction, whereas ninety-eight recipients received consecutive-day ATLG induction.

### 2.3. Immunosuppressive Protocol

All recipients received induction therapy with rabbit-derived anti-T lymphocyte globulin (ATLG; Grafalon^®^, Neovii Biotech, Graefelfing, Germany). ATLG treatment was initiated at approximately 2.0–2.5 mg/kg/day. Subsequent doses were individualized according to daily peripheral CD3-positive T-cell and lymphocyte counts until the institutional targets for peripheral lymphocyte depletion were achieved. For each recipient, cumulative ATLG exposure was calculated as the total administered dose divided by recipient body weight and expressed as mg/kg body weight. Maintenance immunosuppression consisted of tacrolimus, mycophenolate mofetil (1000 mg twice daily), and corticosteroids according to the institutional kidney transplantation protocol. The corticosteroid regimen consisted of intravenous methylprednisolone at 1000 mg on the day of transplantation, followed on successive days by 500 mg, 250 mg, 160 mg, 80 mg, and 40 mg, respectively. On postoperative day 7, patients were transitioned to oral corticosteroid therapy at 20 mg/day. This dose was maintained during the first month and subsequently tapered by 5 mg every two weeks. The institutional target for peripheral CD3+ T-cell depletion was 20–50 cells/µL. Tacrolimus was initiated on the morning after transplantation in all recipients, irrespective of the ATLG administration schedule. Tacrolimus dosing was subsequently individualized according to trough concentrations, with target levels of 10–12 ng/mL during the first post-transplant month, 8–10 ng/mL through month 3, and approximately 8 ng/mL thereafter during the remainder of the first post-transplant year; these targets were applied to both treatment groups.

All recipients received routine CMV prophylaxis after transplantation. CMV monitoring was performed at postoperative month 3 and, if negative, every 3 months thereafter during the first post-transplant year. In recipients with CMV positivity, viral load was monitored weekly while positivity persisted. BK polyomavirus (BKV) monitoring was performed monthly during the first 6 months after transplantation and every 3 months thereafter until the end of the first post-transplant year.

### 2.4. Data Collection

Recipient variables included age, sex, body mass index, dialysis modality, dialysis duration, panel reactive antibody (PRA) class I positivity, and PRA class II positivity. Donor variables included donor age, sex, and body mass index. Transplant-related variables included HLA mismatch score, warm ischemia time, cold ischemia time, postoperative red blood cell transfusion requirement, cumulative ATLG dose (mg), cumulative ATLG exposure (mg/kg body weight), and serum creatinine concentrations. Clinical follow-up variables included delayed graft function, biopsy-proven acute rejection, BK virus positivity, cytomegalovirus (CMV) positivity, bacterial infection during the first post-transplant year, graft loss, and patient survival.

### 2.5. Study Outcomes

The primary outcome was the occurrence of at least one clinically documented bacterial infection requiring antimicrobial treatment during the first 12 months after transplantation. Secondary outcomes included biopsy-proven acute rejection, delayed graft function, BK virus positivity, CMV positivity, serum creatinine concentrations, graft loss, and patient survival. Outcome definitions and diagnostic criteria were predefined and applied consistently to both treatment groups throughout the study period. No changes in the institutional diagnostic approach relevant to these outcomes occurred during the study period. Bacterial infection was evaluated using a fixed 12-month observation window for all recipients. In contrast, biopsy-proven acute rejection, graft loss, and all-cause mortality were recorded throughout each recipient’s entire available follow-up period. Acute rejection was diagnosed by renal allograft biopsy according to the Banff classification. Graft loss was defined as loss of renal allograft function requiring reinstitution of any form of renal replacement therapy. Delayed graft function was defined as the requirement for dialysis within the first week after transplantation. Zero-time and routine protocol (surveillance) graft biopsies were not performed. Renal allograft biopsies were performed only when clinically indicated, primarily in recipients with suspected graft dysfunction.

### 2.6. Statistical Analysis

Statistical analyses were performed using IBM SPSS Statistics version 21 (IBM Corp., Armonk, NY, USA). The distribution of continuous variables was assessed using the Kolmogorov–Smirnov test. Normally distributed variables are presented as mean ± standard deviation and were compared using the independent-samples *t* test. Non-normally distributed variables are presented as median (interquartile range) and were compared using the Mann–Whitney *U* test. Categorical variables are presented as number (percentage) and were compared using Pearson’s chi-square test or Fisher’s exact test, as appropriate. To identify variables independently associated with bacterial infection during the first post-transplant year, multivariable logistic regression analysis was performed. Variables considered clinically relevant based on previous literature or demonstrating a potential association in univariable analyses were entered into the multivariable model. Results are presented as adjusted odds ratios (ORs) with 95% confidence intervals (95% CIs). Propensity score matching was not performed because matching would have reduced the available sample size and number of outcome events in this relatively small cohort; potential confounding was instead addressed using multivariable logistic regression. Nevertheless, residual confounding related to the nonrandomized, time-dependent allocation cannot be excluded.

Because the institutional induction protocol changed during the study period, overall follow-up duration differed between treatment groups. To minimize potential bias arising from unequal follow-up for the primary outcome, bacterial infection was evaluated using an identical 12-month observation period for all recipients. Biopsy-proven acute rejection, graft loss, and all-cause mortality were recorded over the entire available follow-up and are presented as crude event proportions. Because event-specific timing was not available in the analytical dataset, time-to-event analyses could not be performed for these secondary outcomes; therefore, comparisons of these outcomes should be interpreted in the context of unequal follow-up duration. One recipient had missing warm ischemia time data and was therefore excluded from the multivariable logistic regression analysis. No imputation was performed for missing data. A two-sided *p*-value < 0.05 was considered statistically significant.

## 3. Results

### 3.1. Study Population

A total of 195 adult recipients who underwent first living-donor kidney transplantation during the study period met the eligibility criteria and were included in the analysis. According to the institutional induction protocol in effect at the time of transplantation, 98 recipients received consecutive-day anti-T lymphocyte globulin (ATLG) induction, whereas 97 recipients transplanted after the protocol modification received alternate-day ATLG induction. All 98 recipients (100%) in the consecutive-day group received ATLG according to the consecutive-day institutional schedule, and all 97 recipients (100%) in the alternate-day group received ATLG according to the alternate-day institutional schedule; no crossover between administration schedules occurred. A schematic overview of patient allocation and the principal study findings is presented in Figure 1.

Because the institutional induction protocol changed during the study period, recipients treated with alternate-day ATLG had a significantly shorter follow-up duration than those treated with the consecutive-day protocol (25 [18–30] vs. 42 [37–47] months, *p* < 0.001). Bacterial infection was assessed over the same predefined 12-month period in both groups, whereas biopsy-proven acute rejection, graft loss, and all-cause mortality were recorded throughout the entire available follow-up.

### 3.2. Baseline Characteristics

Baseline recipient, donor, immunological, and transplant characteristics are summarized in Table 1.

Recipient characteristics were generally well balanced between the two groups. Recipient age (46 [35–57] vs. 43 [35–50.8] years, *p* = 0.150), recipient sex (*p* = 0.508), body mass index (25.6 [22.3–28.7] vs. 24.9 [22.6–27.7] kg/m2, *p* = 0.498), dialysis modality (*p* = 0.390), and dialysis duration (2 [0–12] vs. 3 [0–12.8] months, *p* = 0.473) were comparable between recipients receiving alternate-day and consecutive-day ATLG. The low proportion of recipients receiving peritoneal dialysis before transplantation reflects the relatively low utilization of peritoneal dialysis compared with hemodialysis in our country.

The two groups also showed similar donor characteristics. Donor age (50 [38–57] vs. 45 [38–53.8] years, *p* = 0.404), donor sex (*p* = 1.000), and donor body mass index (27.3 [24.0–30.1] vs. 28.4 [25.3–32.0] kg/m^2^, *p* = 0.061) did not differ significantly.

Regarding immunological characteristics, recipients treated with consecutive-day ATLG had significantly higher rates of PRA class I positivity (25.5% vs. 8.2%, *p* = 0.002) and PRA class II positivity (18.4% vs. 6.2%, *p* = 0.018). Nevertheless, all recipients were undergoing their first kidney transplantation, all received kidneys from related living donors, and none had preformed donor-specific antibodies before transplantation.

Transplant-related variables were largely comparable between groups. HLA mismatch score (*p* = 0.557) and cold ischemia time (*p* = 0.672) were similar. The cumulative ATLG dose was 550 (450–675) mg in the alternate-day group and 525 (400–700) mg in the consecutive-day group (*p* = 0.321). Corresponding cumulative weight-adjusted ATLG exposure was 7.4 (7.1–7.8) mg/kg and 7.2 (5.4–10.2) mg/kg, respectively (*p* = 0.207). However, recipients receiving alternate-day ATLG had significantly longer warm ischemia times (174.5 [154.5–195.0] vs. 160.0 [141.3–184.5] minutes, *p* = 0.010) and required significantly more postoperative red blood cell transfusions (1 [0–2] vs. 0 [0–1] units, *p* = 0.004). At least one unit of red blood cells was administered to 60 of 97 recipients (61.9%) in the alternate-day group and 38 of 98 recipients (38.8%) in the consecutive-day group.

### 3.3. Clinical Outcomes

Clinical outcomes are summarized in Table 2.

Delayed graft function occurred in only one recipient (1.0%), who belonged to the consecutive-day group. Biopsy-proven acute rejection was observed in 2 recipients (2.1%) in the alternate-day group and in 8 recipients (8.2%) in the consecutive-day group; however, the difference was not statistically significant (*p* = 0.100).

BK virus positivity developed in 18 recipients (18.6%) receiving alternate-day ATLG and in 14 recipients (14.3%) receiving consecutive-day ATLG (*p* = 0.446). Similarly, cytomegalovirus positivity occurred in 17 recipients (17.5%) and 12 recipients (12.2%), respectively (*p* = 0.321).

The occurrence of bacterial infection during the first post-transplant year was virtually identical between treatment groups. Forty-nine recipients (50.5%) in the alternate-day group and 49 recipients (50.0%) in the consecutive-day group experienced at least one clinically documented bacterial infection requiring antimicrobial treatment (*p* = 1.000). Figure 2.

During the entire available follow-up, graft loss occurred in four recipients (4.1%) in each group (crude comparison, *p* = 1.000). All-cause mortality during the entire available follow-up was observed in seven recipients (7.2%) treated with alternate-day ATLG and four recipients (4.1%) treated with consecutive-day ATLG (crude comparison, *p* = 0.372). Because follow-up duration differed significantly between groups and event-specific timing was unavailable, these crude proportions should not be interpreted as time-adjusted comparisons.

### 3.4. Renal Function

Renal allograft function remained stable throughout follow-up in both treatment groups.

Median serum creatinine concentrations were comparable at all predefined assessment time points, including postoperative week 1 (1.05 vs. 1.15 mg/dL, *p* = 0.435), month 1 (1.19 vs. 1.31 mg/dL, *p* = 0.065), month 6 (1.30 vs. 1.34 mg/dL, *p* = 0.504), year 1 (1.29 vs. 1.24 mg/dL, *p* = 0.526), and the last available follow-up visit (1.23 vs. 1.32 mg/dL, *p* = 0.372), indicating similar short- and intermediate-term graft function regardless of the ATLG administration schedule.

### 3.5. Histopathological Findings

Indication biopsies were performed in recipients with clinical suspicion of graft dysfunction. Histopathological diagnoses included acute T-cell-mediated rejection, antibody-mediated rejection, BK virus nephropathy, calcineurin inhibitor toxicity, recurrent focal segmental glomerulosclerosis, acute tubular injury, transplant glomerulopathy, and chronic rejection. The distribution of biopsy findings did not demonstrate a clinically meaningful difference between treatment groups. Zero-time and routine protocol biopsies were not performed during the study period.

### 3.6. Multivariable Logistic Regression Analysis

Multivariable logistic regression analysis was performed to identify variables independently associated with bacterial infection during the first post-transplant year. Recipient demographic characteristics, immunological variables, donor characteristics, ischemia times, cumulative ATLG exposure (mg/kg body weight), and ATLG administration schedule were entered into the regression model. After multivariable adjustment, ATLG administration schedule was not independently associated with bacterial infection (adjusted OR 1.21, 95% CI 0.64–2.30; *p* = 0.560). Likewise, cumulative ATLG exposure expressed as mg/kg body weight was not independently associated with bacterial infection (adjusted OR 1.10, 95% CI 0.98–1.24; *p* = 0.101). Recipient body mass index remained the only independent predictor of bacterial infection (adjusted OR 1.08, 95% CI 1.00–1.15; *p* = 0.041). PRA class II positivity showed a trend toward an increased risk of bacterial infection but did not reach statistical significance (adjusted OR 2.76, 95% CI 0.89–8.51; *p* = 0.077). No independent associations were identified for recipient age, recipient sex, PRA class I positivity, HLA mismatch score, donor age, donor body mass index, dialysis duration, warm ischemia time, or cold ischemia time (Table 3).

## 4. Discussion

The objective of this study was to determine whether alternate-day versus consecutive-day ATLG administration was associated with differences in clinical outcomes in first living-donor kidney transplant recipients at low immunological risk when cumulative weight-adjusted ATLG exposure was accounted for. In accordance with this objective, no difference was observed in the primary outcome of bacterial infection during the first post-transplant year, and the ATLG administration schedule was not independently associated with bacterial infection after multivariable adjustment. No statistically significant crude between-group differences were detected in the evaluated secondary outcomes. However, biopsy-proven acute rejection, graft loss, and mortality were recorded throughout the available follow-up and should be interpreted in the context of unequal follow-up duration. Thus, the present findings are hypothesis-generating and do not establish equivalence between the two administration schedules.

These findings should not be interpreted as demonstrating superiority of alternate-day administration or a cost-effectiveness advantage. Rather, they support the interpretation that, within an individualized depletion-guided strategy, cumulative immunosuppressive exposure and the achieved pharmacodynamic effect may be more clinically relevant than calendar spacing of individual ATLG doses. The present retrospective comparison therefore provides hypothesis-generating real-world evidence regarding scheduling flexibility in a carefully selected low-risk population.

Although ATLG has become an established induction agent in kidney transplantation, previous studies have predominantly focused on cumulative dose optimization, comparisons between induction agents, or dose-reduction strategies rather than on the interval between individual ATLG administrations [6,7,9]. Consequently, evidence specifically evaluating whether administration timing independently influences clinical outcomes remains limited [10]. To our knowledge, this is among the first studies specifically examining alternate-day versus consecutive-day ATLG administration while accounting for cumulative weight-adjusted ATLG exposure in a homogeneous cohort of first living-donor kidney transplant recipients with low immunological risk.

Our findings are consistent with growing evidence indicating that the overall intensity of immunosuppression, rather than the temporal spacing of individual ATLG doses, is the principal determinant of clinical outcomes after lymphocyte-depleting induction therapy [11]. Contemporary studies increasingly support individualized ATLG dosing based on recipient body weight and clinical characteristics, demonstrating satisfactory graft outcomes while maintaining acceptable rates of infectious complications across a broad range of cumulative mg/kg exposures [12]. Similarly, recent consensus recommendations advocate individualized ATLG administration according to recipient immunological risk and cumulative exposure rather than fixed dosing strategies [13].

The biological plausibility of these findings is supported by the pharmacodynamic properties of ATLG. Unlike monoclonal antibodies with relatively predictable pharmacokinetics, polyclonal lymphocyte-depleting antibodies induce sustained depletion of circulating T lymphocytes that persists well beyond the administration of individual doses [14]. Consequently, extending the interval between doses by one or two days would not be expected to substantially alter the overall magnitude or duration of lymphocyte depletion provided that cumulative exposure remains comparable. This mechanism offers a plausible explanation for the absence of clinically meaningful differences in rejection, infectious complications, graft function, or survival observed in the present study.

The homogeneous design of the present cohort is relevant when interpreting these findings. By restricting inclusion to first related living-donor kidney transplant recipients with low immunological risk, negative donor-specific antibodies, and standardized tacrolimus-based maintenance immunosuppression, we reduced several major sources of clinical heterogeneity that have complicated comparisons in previous studies [11,15].

An important observation in the present study was that the significantly higher prevalence of PRA class I and class II positivity in the consecutive-day group was not accompanied by an increased incidence of biopsy-proven acute rejection. Although pre-transplant sensitization is an established immunological risk factor, all recipients in our cohort were negative for donor-specific antibodies and underwent first living-donor kidney transplantation. Therefore, the observed differences in PRA positivity most likely reflected low-level sensitization rather than clinically relevant alloimmune risk. These findings suggest that, within a carefully selected low-risk population receiving standardized maintenance immunosuppression, modest differences in PRA positivity may not necessarily translate into inferior short-term clinical outcomes.

Biopsy-proven acute rejection occurred infrequently in both groups and did not differ significantly according to the ATLG administration schedule. Although the numerical incidence was lower in recipients receiving alternate-day ATLG, the small number of rejection events limits the ability to detect modest between-group differences. Comparable rejection rates have been reported in studies evaluating individualized or reduced-dose lymphocyte-depleting induction strategies in selected low-risk kidney transplant recipients [16]. Collectively, these findings support the concept that maintenance of adequate cumulative immunosuppressive exposure is more important than the precise timing of individual ATLG doses for preventing acute rejection.

The primary endpoint of our study was the occurrence of bacterial infection during the first post-transplant year. Importantly, bacterial infection occurred at virtually identical frequencies in the alternate-day and consecutive-day groups, and the administration schedule was not independently associated with infection after adjustment for potential confounding variables. These findings are clinically relevant because temporary postponement of ATLG administration is frequently required in daily practice owing to leukopenia, thrombocytopenia, infusion-related adverse reactions, or perioperative clinical conditions [17]. Our results suggest that such modifications in administration timing may be performed without increasing infectious complications, provided that cumulative weight-adjusted ATLG exposure is preserved.

Multivariable logistic regression analysis further strengthened these observations. Neither the ATLG administration schedule nor cumulative weight-adjusted ATLG exposure independently predicted bacterial infection. Instead, recipient body mass index emerged as the only independent predictor. Obesity has consistently been associated with impaired wound healing, prolonged operative duration, altered innate and adaptive immune responses, and increased susceptibility to postoperative urinary tract and surgical-site infections after kidney transplantation [18,19]. Our findings therefore support the growing evidence that recipient-related factors may contribute more substantially to infectious risk than modest variations in induction therapy scheduling.

No significant differences were observed between the study groups with respect to BK virus or CMV positivity. These findings are consistent with previous reports demonstrating that opportunistic viral infections are primarily influenced by the cumulative intensity of immunosuppression and the recipient’s net state of immunosuppression rather than by individual induction protocols [20,21]. The concept of the “net state of immunosuppression” has become increasingly important in transplantation medicine because infection risk reflects the combined effects of induction therapy, maintenance immunosuppression, recipient characteristics, and environmental exposures rather than any single immunosuppressive agent alone.

Similarly, renal allograft function remained stable throughout follow-up, and serum creatinine concentrations were comparable between the alternate-day and consecutive-day groups at all predefined assessment time points. Crude graft loss and mortality proportions did not differ significantly between groups; however, unlike the primary bacterial infection outcome, these events were recorded throughout the entire available follow-up. Because recipients receiving alternate-day ATLG had a shorter overall follow-up after the institutional protocol change, these crude secondary-outcome comparisons are not adjusted for time at risk and should therefore be interpreted cautiously. The primary endpoint, by contrast, was evaluated using an identical 12-month observation period for all recipients.

Several strengths of the present study should be acknowledged. The deliberately homogeneous population of first related living-donor kidney transplant recipients with low immunological risk, negative donor-specific antibodies, and standardized tacrolimus-, mycophenolate mofetil-, and corticosteroid-based maintenance immunosuppression reduced the potential influence of baseline clinical and immunological heterogeneity. In addition, cumulative ATLG exposure was evaluated as total administered dose per kilogram of recipient body weight, reflecting individualized weight-adjusted dosing. Finally, the primary outcome was assessed using an identical 12-month observation period for all recipients, minimizing bias arising from unequal overall follow-up duration for the principal study endpoint.

Several limitations should also be considered. First, the retrospective single-center design limits the ability to establish causal relationships and may reduce the generalizability of the findings. Second, because treatment allocation was determined by an institutional protocol change rather than randomization, residual confounding cannot be completely excluded despite multivariable adjustment. Third, the relatively small number of biopsy-proven acute rejection events may have limited the statistical power to detect modest between-group differences. Fourth, although bacterial infection was evaluated within the same predefined 12-month period in all recipients, biopsy-proven acute rejection, graft loss, and all-cause mortality were recorded throughout the entire available follow-up. Because overall follow-up was significantly shorter in the alternate-day group and event-specific timing was not available in the analytical dataset, time-to-event analyses could not be performed for these secondary outcomes; their crude between-group comparisons should therefore be interpreted cautiously. In addition, although cumulative absolute and weight-adjusted ATLG doses were available, the exact treatment duration (i.e., the interval from the first to the final ATLG dose) was not recorded as a separate variable in the retrospective analytical dataset and could not be reliably reconstructed. The dataset also did not include serial CD3+ T-cell measurements sufficient to determine the time to target T-cell depletion or systematically collected hematological adverse-event data. Therefore, potential between-group differences in depletion kinetics and hematological toxicity could not be assessed. Length of hospital stay and healthcare-resource utilization were not available in the analytical dataset; therefore, the potential impact of alternate-day administration on hospitalization duration or healthcare burden could not be assessed. Propensity score matching was not performed, and multivariable adjustment cannot eliminate all bias arising from the nonrandomized, time-dependent allocation.

Despite these limitations, our study provides clinically relevant, hypothesis-generating evidence addressing a question that has received little attention in the existing literature. While previous investigations have largely concentrated on cumulative ATLG dose, comparisons between induction agents, or dose-reduction strategies, the potential clinical impact of extending the interval between individual ATLG doses has remained uncertain. Our findings suggest that modest variation in administration timing may be feasible in carefully selected first living-donor kidney transplant recipients with low immunological risk when cumulative weight-adjusted ATLG exposure is maintained. An important next step will be to determine whether specific recipient subgroups derive differential benefit or risk from alternate-day versus consecutive-day dosing. Prospective studies should therefore evaluate populations in whom dosing interval may be more clinically relevant, including recipients at higher immunological risk, patients with impaired early graft function, recipients in whom calcineurin inhibitor initiation is intentionally delayed, and patients at increased risk of infectious or hematological complications. Such studies should incorporate standardized follow-up and time-to-event analyses for rejection, graft loss, and patient survival.

## 5. Conclusions

In this retrospective cohort of first living-donor kidney transplant recipients at low immunological risk, no statistically significant difference was detected between alternate-day and consecutive-day ATLG administration in the primary outcome of bacterial infection during the first post-transplant year. After multivariable adjustment, the ATLG administration schedule was not independently associated with bacterial infection. These findings should not be interpreted as establishing equivalence or non-inferiority between the two regimens. Rather, they provide hypothesis-generating evidence that modest flexibility in ATLG dose spacing may be feasible in carefully selected recipients when cumulative weight-adjusted exposure is maintained. Because biopsy-proven acute rejection, graft loss, and mortality were assessed over unequal overall follow-up periods, conclusions regarding these secondary outcomes should remain cautious. Prospective multicenter studies with standardized follow-up and appropriate time-to-event analyses are required to confirm these observations.

## Figures and Tables

**Figure 1:**
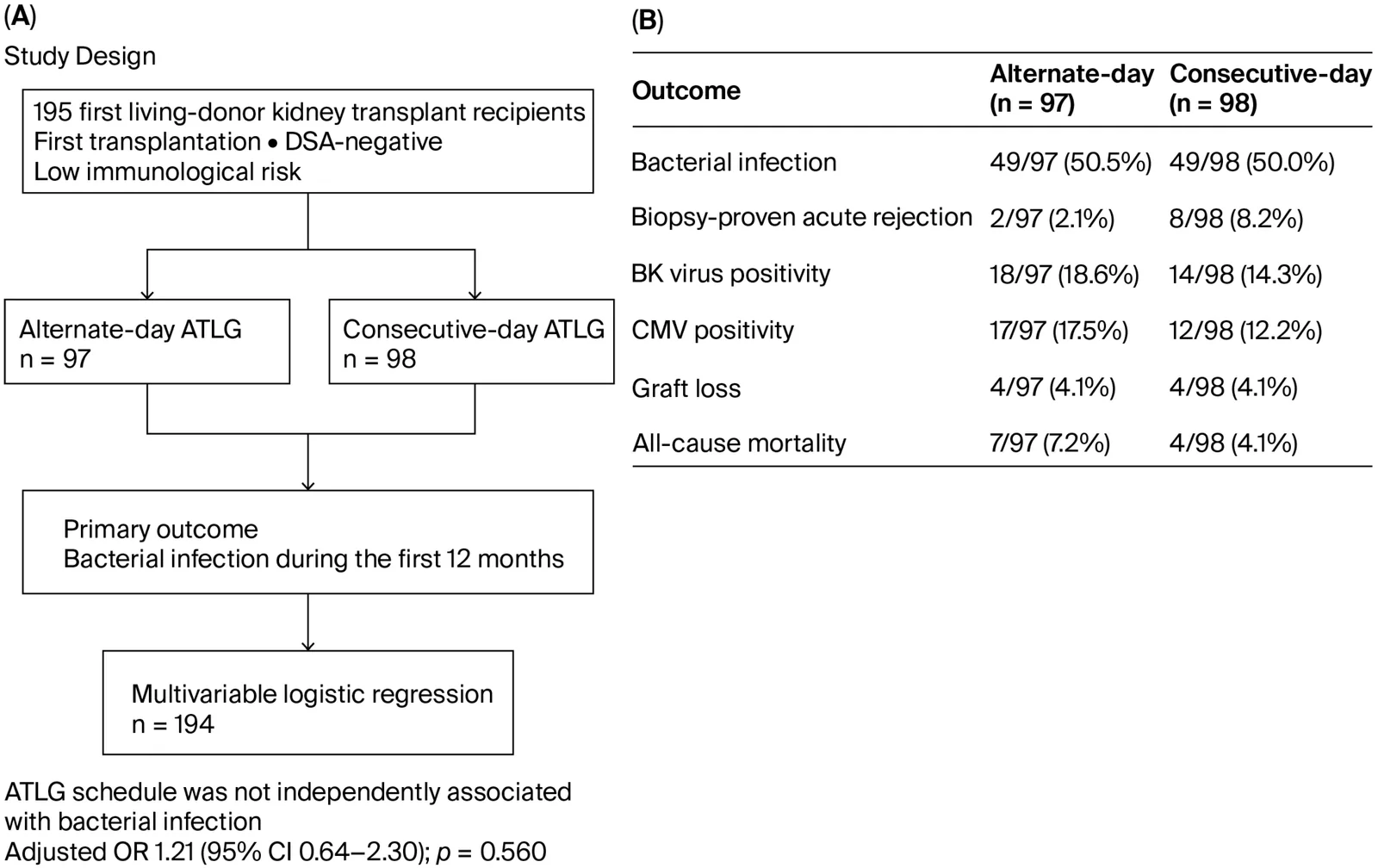
Study overview and principal findings. (**A**) illustrates the study design, patient allocation, primary outcome assessment, and (**B**) illustrates major clinical outcomes following alternate-day and consecutive-day anti-T lymphocyte globulin (ATLG) induction. No statistically significant crude between-group differences were observed for the reported clinical outcomes; bacterial infection was assessed during the first 12 months, whereas rejection, graft loss, and mortality were recorded throughout the available follow-up. In multivariable analysis, alternate-day ATLG administration was not independently associated with bacterial infection (adjusted OR, 1.21; 95% CI, 0.64–2.30; *p* = 0.560).

**Figure 2:**
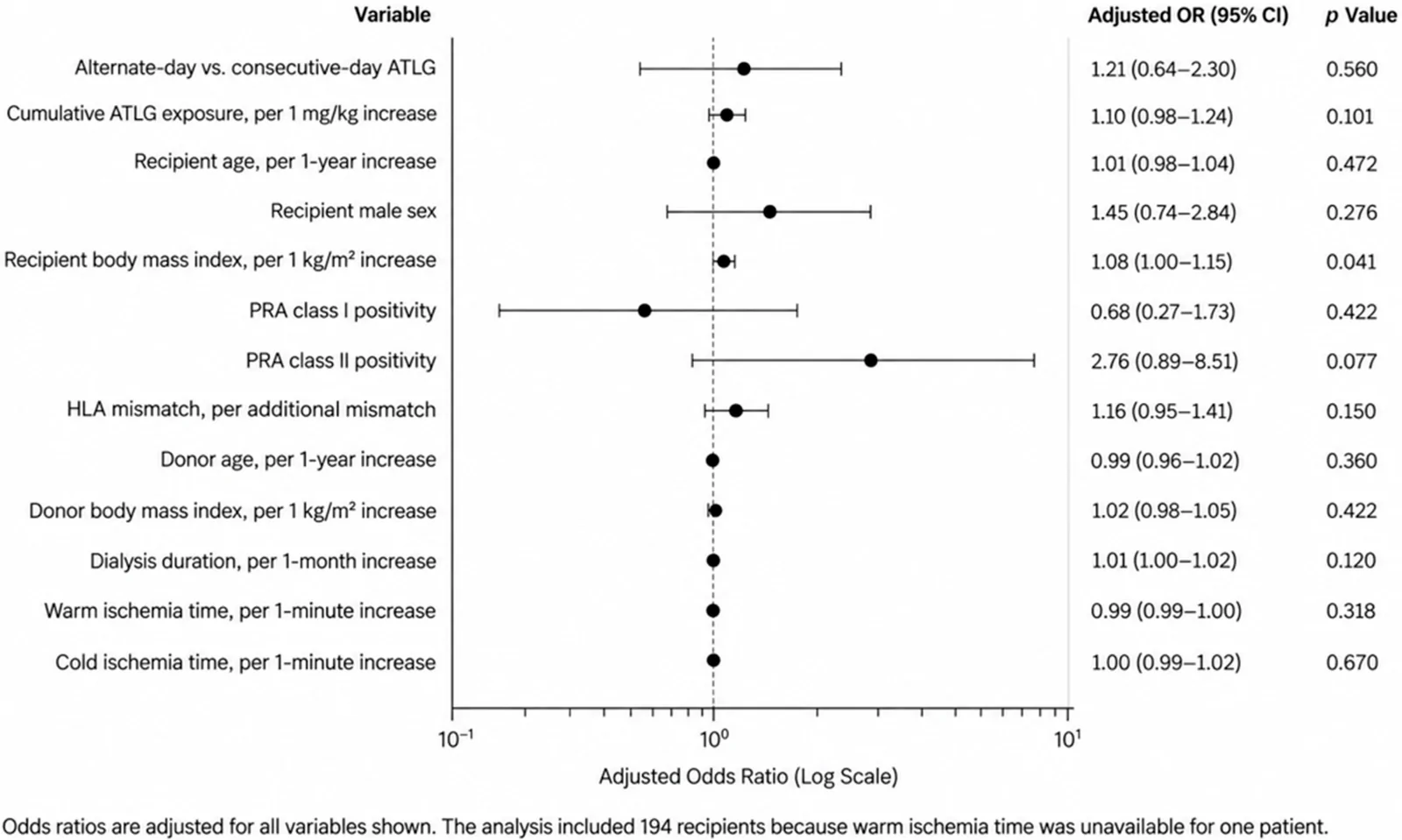
Forest plot showing the adjusted odds ratios (ORs) with 95% confidence intervals for variables included in the multivariable logistic regression model evaluating factors associated with bacterial infection during the first post-transplant year.

**Table 1:** Baseline recipient, donor, immunological and transplant characteristics.

Variable	Alternate-Day ATLG (n = 97)	Consecutive-Day ATLG (n = 98)	*p* Value
Recipient characteristics			
Age, years	46 (35–57)	43 (35–50.8)	0.150
Recipient male sex, n (%)	63 (64.9)	69 (70.4)	0.508
Body mass index, kg/m^2^	25.6 (22.3–28.7)	24.9 (22.6–27.7)	0.498
Pre-transplant renal replacement therapy, n (%)			0.390
Preemptive transplantation	42 (43.3)	37 (37.8)	
Hemodialysis	52 (53.6)	60 (61.2)	
Peritoneal dialysis	3 (3.1)	1 (1.0)	
Dialysis duration, months	2 (0–12)	3 (0–12.8)	0.473
Immunological characteristics			
PRA class I positive, n (%)	8 (8.2)	25 (25.5)	**0.002**
PRA class II positive, n (%)	6 (6.2)	18 (18.4)	**0.018**
Donor characteristics			
Donor age, years	50 (38–57)	45 (38–53.8)	0.404
Male donor, n (%)	41 (42.3)	41 (41.8)	1.000
Donor body mass index, kg/m^2^	27.3 (24.0–30.1)	28.4 (25.3–32.0)	0.061
Transplant characteristics			
HLA mismatch	4 (3–5)	4 (3–5)	0.557
Warm ischemia time, min	174.5 (154.5–195.0)	160.0 (141.3–184.5)	**0.010**
Cold ischemia time, min	64 (56–74)	62 (55–72.8)	0.672
Postoperative red blood cell transfusion, units	1 (0–2)	0 (0–1)	**0.004**
Cumulative ATLG dose, mg	550 (450–675)	525 (400–700)	0.321
Cumulative ATLG exposure, mg/kg body weight	7.4 (7.1–7.8)	7.2 (5.4–10.2)	0.207
Adherence to assigned ATLG administration schedule, n (%)	97 (100)	98 (100)	—
Follow-up duration, months	25 (18–30)	42 (37–47)	**<0.001**

Note: Continuous variables are presented as median (interquartile range), and categorical variables as number (%). Continuous variables were compared using the Mann–Whitney U test. Categorical variables were compared using the χ^2^ test or Fisher’s exact test, as appropriate. Bold values indicate statistical significance (*p* < 0.05).

**Table 2:** Clinical outcomes according to ATLG administration schedule.

Outcome	Alternate-Day ATLG (n = 97)	Consecutive-Day ATLG (n = 98)	*p* Value
Delayed graft function, n (%)	0 (0.0)	1 (1.0)	1.000
Biopsy-proven acute rejection, n (%)	2 (2.1)	8 (8.2)	0.100
BK virus positivity, n (%)	18 (18.6)	14 (14.3)	0.446
Cytomegalovirus positivity, n (%)	17 (17.5)	12 (12.2)	0.321
Bacterial infection during the first post-transplant year, n (%)	**49 (50.5)**	**49 (50.0)**	**1.000**
Graft loss, n (%)	4 (4.1)	4 (4.1)	1.000
All-cause mortality, n (%)	7 (7.2)	4 (4.1)	0.372

Note: Values are presented as number (%). Comparisons were performed using Pearson’s χ^2^ test or Fisher’s exact test, as appropriate. Bacterial infection was assessed during the first 12 months after transplantation, whereas biopsy-proven acute rejection, graft loss, and all-cause mortality were recorded throughout the entire available follow-up. Because follow-up duration differed between groups, graft loss and mortality comparisons represent crude event proportions and are not time-adjusted. Bold values indicate statistical significance (*p* < 0.05).

**Table 3:** Multivariable logistic regression analysis of factors associated with bacterial infection during the first post-transplant year.

Variable	Adjusted OR	95% CI	*p* Value
Alternate-day vs. consecutive-day ATLG administration	1.21	0.64–2.30	0.560
Cumulative ATLG exposure, per 1 mg/kg increase	1.10	0.98–1.24	0.101
Recipient age, per 1-year increase	1.01	0.98–1.04	0.472
Recipient male sex	1.45	0.74–2.84	0.276
Recipient body mass index, per 1 kg/m^2^ increase	**1.08**	**1.00–1.15**	**0.041**
PRA class I positivity	0.68	0.27–1.73	0.422
PRA class II positivity	2.76	0.89–8.51	0.077
HLA mismatch, per additional mismatch	1.16	0.95–1.41	0.150
Donor age, per 1-year increase	0.99	0.96–1.02	0.360
Donor body mass index, per 1 kg/m^2^ increase	1.02	0.98–1.05	0.422
Dialysis duration, per 1-month increase	1.01	1.00–1.02	0.120
Warm ischemia time, per 1 min increase	0.99	0.99–1.00	0.318
Cold ischemia time, per 1 min increase	1.00	0.99–1.02	0.670

Abbreviations: ATLG, anti-T lymphocyte globulin; CI, confidence interval; HLA, human leukocyte antigen; OR, odds ratio; PRA, panel reactive antibody. Note: The dependent variable was the occurrence of at least one clinically documented bacterial infection during the first 12 months after transplantation. The analysis included 194 recipients because warm ischemia time was unavailable for one patient. Bold values indicate statistical significance (*p* < 0.05).

## Data Availability

The datasets generated and/or analyzed during the current study are not publicly available because they contain identifiable clinical information.

## Abbreviations

ATLG	Anti-T lymphocyte globulin
CMV	Cytomegalovirus
BK	BK polyomavirus
DSA	Donor-specific antibody
PRA	Panel reactive antibody
HLA	Human leukocyte antigen
OR	Odds ratio
CD3	Cluster of differentiation 3-positive T cells
CI	Confidence interval
IBM	International Business Machines
BMI	Body mass index
SPSS	Statistical Package for the Social Sciences
n	Number
*p*	*p* value / probability value
mg	Milligram
kg	Kilogram
mg/kg	Milligrams per kilogram
kg/m^2^	Kilograms per square meter
ng/mL	Nanograms per milliliter
mg/dL	Milligrams per deciliter
cells/µL	Cells per microliter
min	Minute(s)
